# Supplementary material for: Tyrosine Is a Booster of Leucine-Induced Muscle Anabolic Response
Source: Nutrients. 2023 Dec 26;16(1):84. doi: 10.3390/nu16010084 (PMC10780460; doi:10.3390/nu16010084)
Supplement: Supplementary file 1 [file nutrients-16-00084-s001.zip › nutrients-2749459-supplementary materials.pdf]

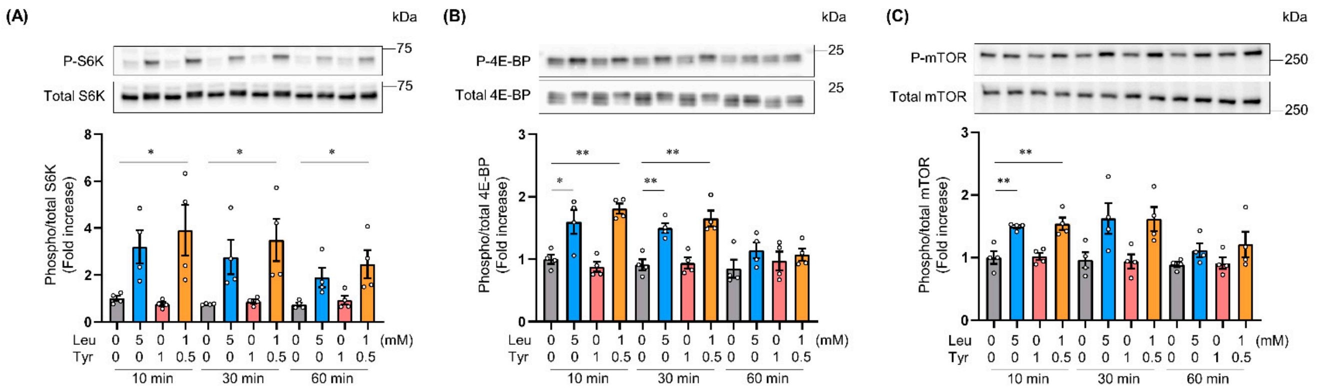

Supplemental Figure S1. Time course response of leucine and tyrosine in the anabolic signaling pathway in C2C12 myoblasts

Phosphorylation of S6K (Thr389) (A), 4E-BP (Thr37/46) (B), and mTOR (Ser2448) (C) 10–60 min after stimulation with Leu and/or Tyr in C2C12 myoblasts on day 1 post-differentiation (n = 4). All data is shown as fold change to Ctrl (0 mM Leu and 0 mM Tyr) at 10 min. Data are presented as the mean ± SEM. Circles represent individual values. *$p < 0.05$, **$p < 0.01$ vs. Ctrl as determined by one-way ANOVA.

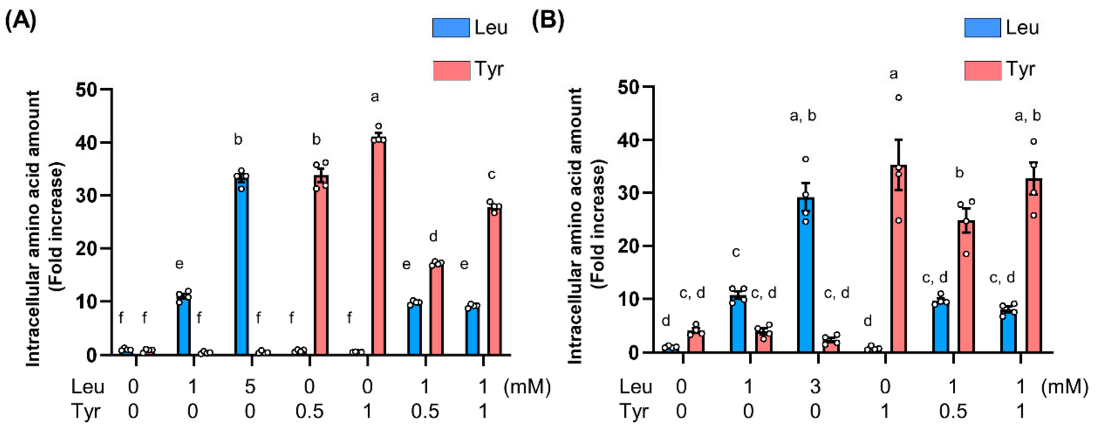

Supplemental Figure S2. Measurement of intracellular free amino acid levels following exposure to leucine and tyrosine

Intracellular free Leu and Tyr levels in (A) C2C12 myoblasts and (B) isolated muscle (EDL) were determined by LC-MS/MS. Measurements of the isolated muscle were corrected for the weight of the muscle brought in. All values are presented relative to the amount of Leu in the control group. Data are presented as the mean ± SEM (n = 4). Circles represent individual values. Different letters (a–f) indicated significant differences ($p < 0.05$) as determined by one-way ANOVA.

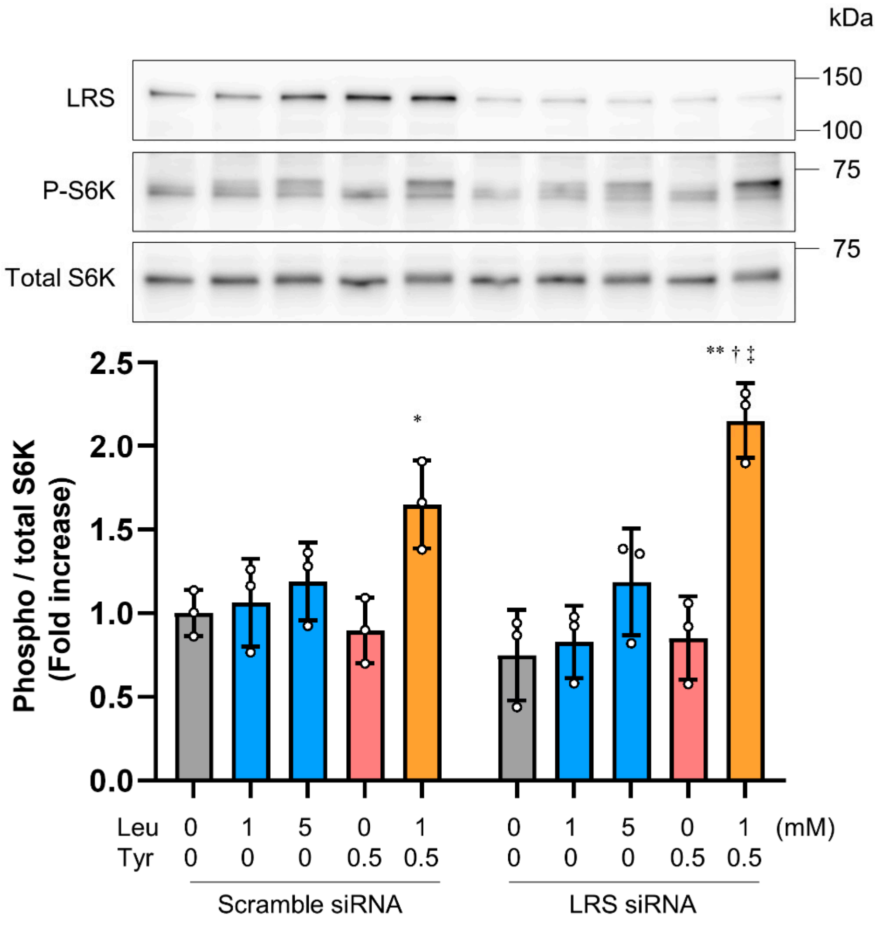

Supplemental Figure S3. Effect of LRS expression suppression on leucine and tyrosine-induced S6K phosphorylation

S6K phosphorylation (Thr389) in response to 15 min of stimulation with Leu and/or Tyr under downregulated LRS expression by siRNA transfection in C2C12 myoblasts on day 1 post-differentiation. Data are presented as the mean ± SEM (n = 3). Circles represent individual values. *$p < 0.05$ vs. Ctrl (Scr siRNA). ** $p < 0.01$ vs Ctrl (LRS siRNA). † $p < 0.01$ vs. 1 mM Leu (LRS siRNA). ‡ $p < 0.01$ vs. 5 mM Leu (LRS siRNA) as determined by two-way ANOVA.
